# A concise methodology for the estimation of elemental concentration effects on mesoscale cohesion of non-ferrous covalent glasses: The case of Se_(80−*x*)_Ge_(20−*x*)_In_*x*=0,5,10,15_

**DOI:** 10.1016/j.dib.2015.05.024

**Published:** 2015-06-16

**Authors:** Georgios S.E. Antipas

**Affiliations:** School of Mining Engineering and Metallurgy, National Technical University of Athens, Zografou Campus, Athens 15780, Greece

## Abstract

The link between the electronic state and the mesoscale of covalent glasses is not settled. A functional means of addressing the mesoscale is via generalizing glass properties (e.g. such as cohesion) on the basis of atomic clusters. Derivation of the most representative such cluster formations is not streamlined, however. Here, numerical pair correlation and ab initio energetic datasets are presented for the case of amorphous Selenium-rich covalent glasses, which were obtained via a new, concise methodology, relating mesoscopic cohesion to local atomic order and to the system׳s electronic structure. The methodology consisted of selecting clusters on the basis of the variation of atomic environment statistics of total coordination, partial coordination by the matrix element and cluster number density along the radial direction of a Reverse Monte Carlo supercell, the latter attained by fitting total scattering data.

**Table:** 

Subject area	*Materials Science*, *Quantum Chemistry*
More specific subject area	*Covalent glasses*, *short range order*, *condensed matter physics*
Type of data	*Excel spreadsheet*, *flat text data files*
How data was acquired	Fourier transform of total scattering data, reverse Monte Carlo (RMC) fitting of total pair distribution functions, density functional theory relaxation of atomic clusters selected on the basis of atomic coordination distribution of along the radial direction of the RMC supercell.
Data format	*Text and spreadsheets*
Experimental factors	*None*
Experimental features	*None*
Data source location	*Not applicable*
Data accessibility	*Data is with this paper*

**Value of the data**

• The results obtained provided new insight on the relation between mesoscale cohesion and atomic short range ordering. Based on these results, any solute–solvent interaction occurring in amorphous covalent glasses may in principle be interpreted on the basis of atomic clusters.
• Any high-resolution (total scattering) X ray diffraction measurement may be fed to the methodology presented here.
• The methodology itself is a simple, step-by-step procedure for the rational selection of atomic clusters which are most representative of an amorphous alloy mesoscale. The underlying datasets attached in the paper will help implement the methodology for the chalcogenide glass presented and, once the reader is satisfied that their implementation is sound, may then be extended to other glassy systems of choice.

## 1 Experimental design, materials and methods

In the current work, four chalcogenide glasses with nominal atomic compositions described by Se_(80−*x*)_Ge_(20−*x*)_In_*x*=0,5,10,15_ were synthesized in from elemental Se, Ge and In (all elements were of 99.99% purity) by sealing the required quantities in quartz ampoules under a pressure of 10^−3^ Pa and heating at a rate of 2 K/min up to 1273 K under continuous vibration stirring [1]. Then the samples were rapidly quenched in a mixture of water and ice and the resulting glasses were studied by XRD and EXAFS.

### 1.1 Total scattering

Total scattering datasets were obtained by X-Ray diffraction (XRD) and Extended X-Ray Absorption Fine Structure (EXAFS) spectroscopy. The XRD datasets were recorded by a Ge solid-state detector at the BW5 facility in HASYLAB, DESY at incident beam energy of 100 keV with a cross section equal to 4 mm^2^ and appropriate corrections (background, absorption, polarization) were imposed on the resultant data [1]. EXAFS transmission datasets (approximately 1/*e*) were attained with a step size of 0.5 eV in the vicinity of the absorption edge for Ge, Se and In K-edges at the HASYLAB X beamline.

### 1.2 Reverse Monte Carlo fitting

Here, the experimental XRD and EXAFS datasets were fitted by the RMC method via use of the molecular RMC_POT code [2]. In our simulation we retained the minimum interatomic distances (cut-offs) established in [1]. The RMC simulation boxes each contained 3000. The materials׳ total structure factors, *S*(*Q*), were estimated on the basis of the experimental X-ray and neutron scattering intensities attained; The total *S*(*Q*) data were then correlated to the partial structure factors, *S*_*ij*_(*Q*) via the Faber–Ziman formalism [3]. According to the formalism, the atomic weights, *w*_*ij*_, representing the correlation between any two atomic species *i* and *j* during X-ray scattering are first defined as

(1) $$w_{ij}(Q)=(2-\delta_{ij})c_{i}c_{j}\frac{f_{i}(Q)f_{j}(Q)}{{\langle f(Q)\rangle }^{2}}$$

where *Q* is the scattering wavevector, equal to 4*π*sin(*θ*)/*λ*, *θ* is half of the scattering angle, *λ* is the radiation wavelength, *δ*_*ij*_ is the Kronecker delta function, *c*_*i*_ is the molar fraction of the *i*th element in the system and *f*_*i*_ is the element׳s form factor. The system׳s partial structure factors are then related to the experimentally established total *S*(*Q*) via the expression

(2) $$S(Q)=\sum_{i\leq j}w_{ij}(Q)S_{ij}(Q)$$

The Faber–Ziman partial structure factors, *S*_*ij*_(*Q*), are, in turn, linked to the partial pair distribution functions (PDF), *g*_*ij*_(*r*), through the relation

(2′) $$S(Q)=\sum_{i\leq j}w_{ij}(Q)S_{ij}(Q)$$

(3) $$g_{ij}(r)=1+\frac{1}{2\pi 2\rho_{0}r}\int_{0}^{\infty }Q(S_{ij}(Q)-1)\sin(Qr)dQ$$

where *r* is the real space (Cartesian) variable and *ρ*_ο_ is the alloy׳s number density.

### 1.3 Sensitivity analysis of pair correlation statistics

**Fig. 1:**
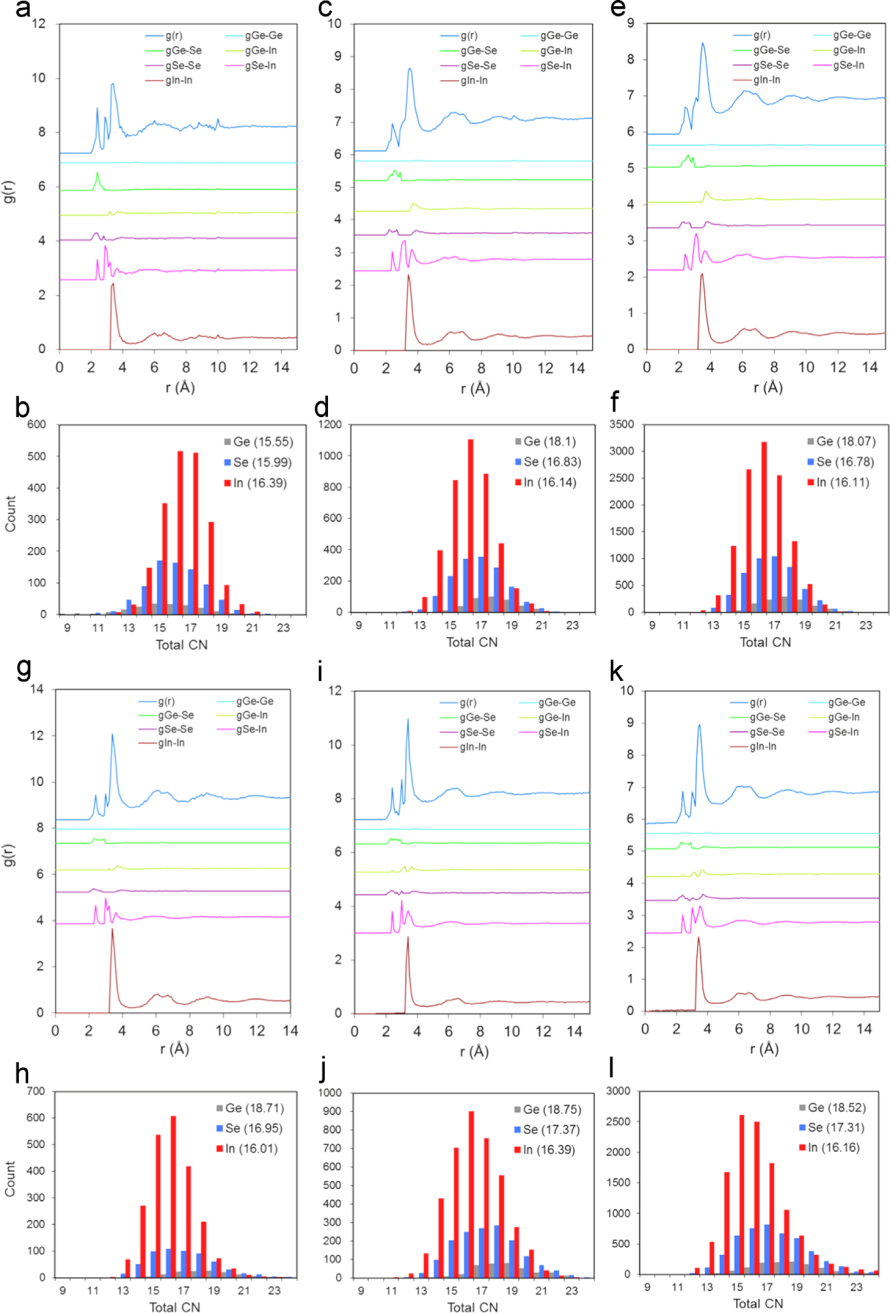
PDF and total cluster coordination (Total CN) as a result of RMC supercell size. For the GeSe_4_In_10_ system: (a), (b) 3000 atoms, (c), (d) 6000 atoms and (e), (f) 18,000 atoms. For the GeSe_4_In_15_ system: (g), (h) 3000 atoms, (i), (j) 6000 atoms and (k), (l) 18,000 atoms. The arithmetic values of CN for each of the models are shown in parentheses.

Inherently, the Metropolis sampling scheme employed in RMC will not place atoms in identical positions in different runs; however the method is expected to produce statistical consistency of average atomic coordination subject to supercell size. The effect of supercell size on the PDF as well as on total cluster coordination is shown in Fig. 1, for two systems of choice: the GeSe_4_In_10_ and GeSe_4_In_15_, each for supercell sizes of 3000, 6000 and 18000 atoms. The choice of these two systems was based on their difficulty to converge; however more solid conclusions may be reached by an exhaustive sensitivity analysis of all eight systems considered. As seen in Fig. 1, RMC shows a fair degree of consistency in the coordination of the matrix element, In, as increasing supercell size in both systems leads to very similar coordination arithmetic values. Equally consistent, however, across the supercell models is a fluctuation of coordination of the solute elements, principally of Ge which is the least abundant, and, hence the element which affects the PDF the least. Additionally, all principal PDF peaks were unaffected by the RMC supercell size. Therefore it is envisaged that upon increasing supercell size, atomic pair correlation statistics for the same system will remain constant if the method is given sufficient time to converge.

**Fig. 2:**
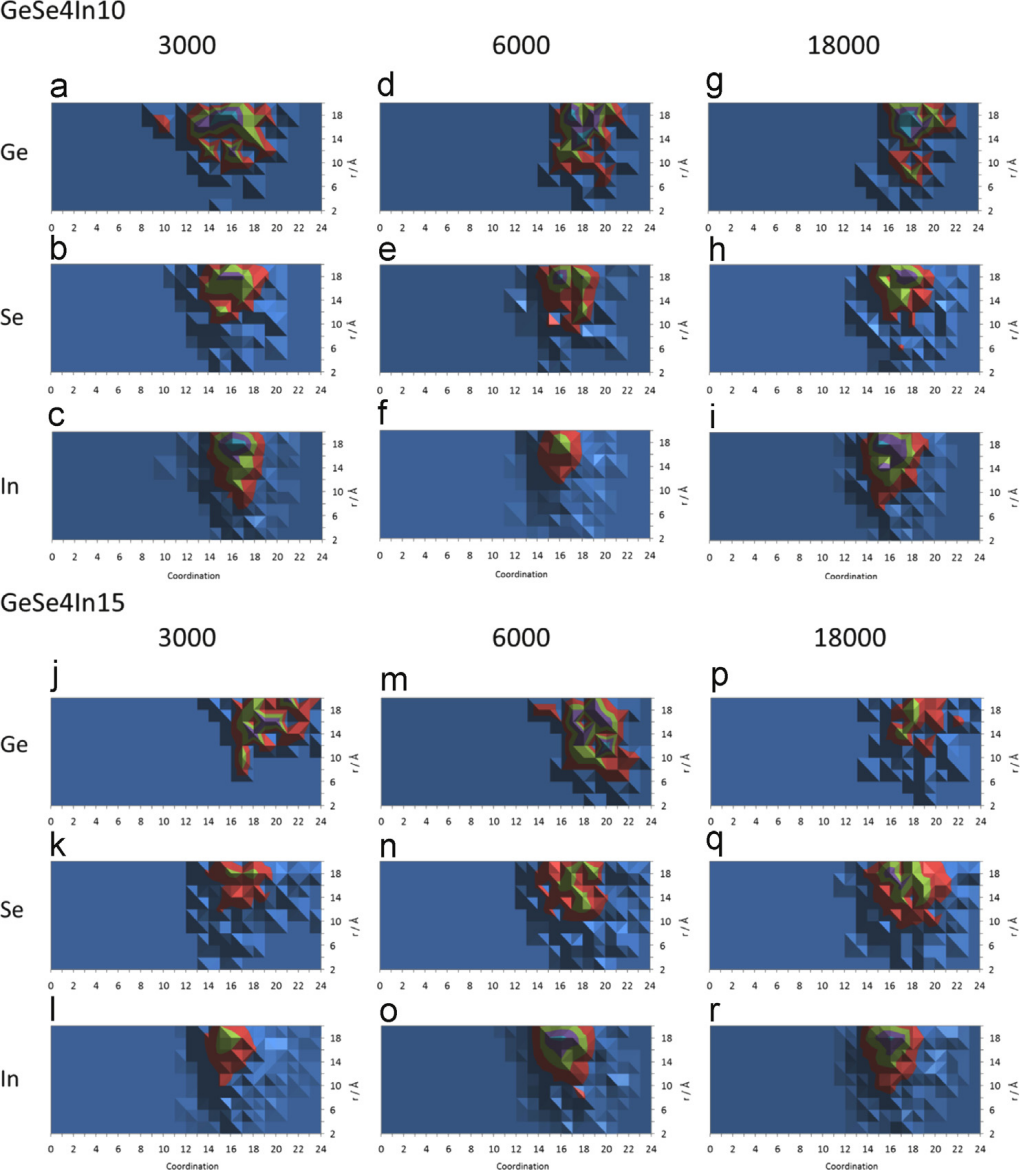
Total coordination distribution of Ge-, Se- and In-centered clusters (shown in sets of three vertical plots each, e.g. (a), (b) and (c)), in respect to their distance (measured from each cluster׳s center atom) from the supercell Cartesian origin. The data depict distributions for the two case systems, GeSe_4_In_10_ (a) to (i) and GeSe_4_In15 (j) to (r) and for each of the two systems the distributions for 3000, 6000 and 18000 atom RMC simulations are shown. The graphs comprise iso-surfaces and the scale of merit are surfaces colored either green or purple which represent coordination peaks, hence the motifs within these regions are more important that their surrounding coordination motifs. For each system, the comparison should be made along a line of graphs, e.g. among (a), (d) and (g), as to whether increasing supercell size results in consistency of the (shape of the) coordination motif. However, arithmetically, as shown in Table 1, the most important coordination motifs are shown to be maintained across supercell sizes.

**Fig. 3:**
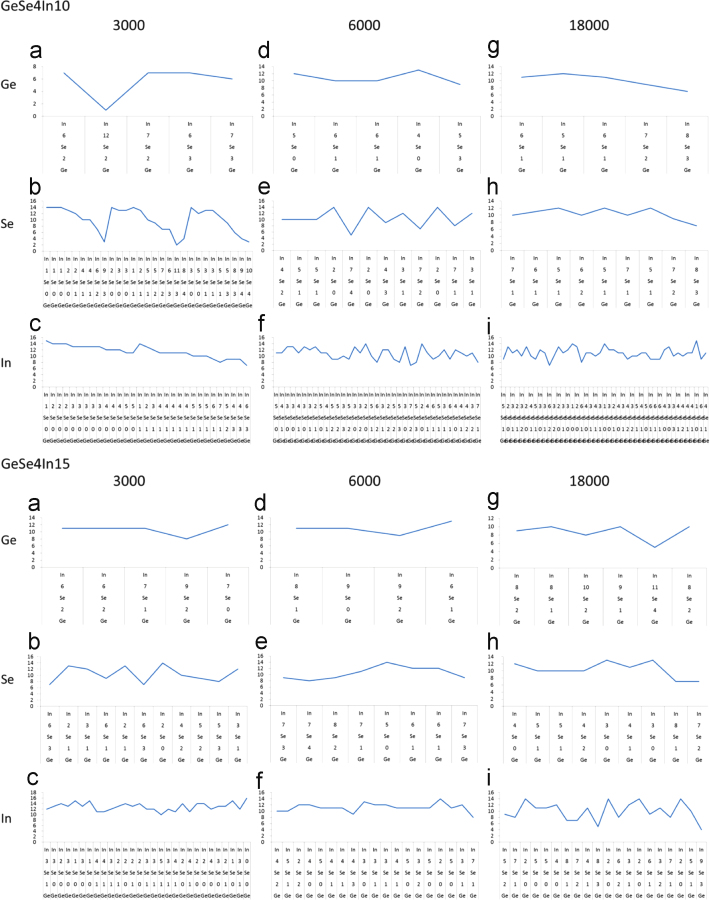
Distribution of Ge, Se and In partial coordination of Ge-, Se- and In-centered clusters (again shown in sets of three vertical plots each, e.g. (a), (b) and (c)), for each of the two case systems and the three supercell sizes for each system. The data represent the number of clusters with different partial coordinations for given total coordinations and distances from the supercell origin as listed in Table 1. For example, plots (a), (b) and (c) are numbers of different clusters in the GeSe_4_In_10_ 3000-atom supercell for which the total coordination is 15 or 16 and lies 18 Å away from the supercell origin. Each line represents the number of In atoms coordinating a Ge-, Se- or In-centered cluster (the center atom species is shown in the far left for each set of plots) for which the rest of the Ge and Se coordinating atoms are fixed and shown in the *X* axis. For example in (a), the first line point represents 7 In atoms coordinating a Ge-centered cluster along with 2 Ge and 6 Se atoms.

Drilling down in terms of the consistency of coordination motifs in respect to supercell size, the total coordination distribution of Ge-, Se- and In-centered clusters in respect to their distance from the supercell׳s origin is shown in Fig. 2. The data suggest that there is, indeed, consistency in the main coordination motifs across supercell size for both systems. The main peaks in each of the iso-surface plots presented in Fig. 2 are listed in Table 1. It is of particular interest to observe that, quite uniformly, the majority of coordination features of interest are located roughly 18 Å away from supercell origins and that the shapes of these motifs are similar across supercell sizes for a given cluster central atom. Altogether, in the province of statistical convergence of the Metropolis sampling scheme, the motifs in Fig. 2 may be declared as consistent across supercell sizes in each of the two case systems. Moreover, the variance of partial cluster coordination for clusters located at the distances listed in Table 1, is shown in Fig. 3; the main point raised in this figure is that there is limited variation in the partial cluster coordination across supercell sizes for the same system. Indicatively, the coordination peak for Ge-centered clusters in the GeSe_4_In_10_ system (see Fig. 2a, d and g) lies at a distance of 18 Å from any supercell origin and comprises, e.g. 15- and 16-fold coordination in the case of the 3000-atom supercell (see Table 1). These clusters are coordinated fairly consistently by six Se and by between eight and twelve In atoms, as may be seen by a comparison of Fig. 3d and g, while there is some deviation of In coordination in the smaller (3000 atom) cluster in Fig. 3a. However, this type of generalization is across all supercells, such that cluster selection on the grounds of the coordination motifs shown in Fig. 2 may be justified.

### 1.4 Cluster selection

Following RMC fitting, a number of Ge-, Se- and In-centered clusters were then chosen as indicative of various positions in the RMC supercell, on the basis of the radial distribution of cluster number density, total average coordination and partial coordination in respect to each element (as already exemplified in Figs. 2 and 3 and Table 1).

### 1.5 Atomistic calculations

Spin unrestricted, DFT calculations were performed with the Amsterdam density functional (ADF) program [4–7] in the GGA BLYP [8,9]/TZ2P level of theory (the TZ2P basis set expands single-electron wavefunctions into uncontracted Slater-type orbitals (STO) comprising a triple-ζ basis set with two sets of polarization functions) for all atoms. Calculations were all-electron for the Ge ([Ar]3d^10^4s^2^4p^2^), Se ([Ar]3d^10^4s^2^4p^4^) and In ([Kr]4d^10^5s^2^5p^1^) structures and they were corrected for relativistic effects using the zero-order regular approximation (ZORA) [10–12], a requirement raised by the presence of In.

In principle, the isolated RMC clusters do not correspond to any particular level of theory; they, hence, had to be relaxed into the BLYP/TZ2P level, treating the case of clusters inclusive of the center׳s second coordination shell, for two scenarios: a) relaxation of the first coordination sphere keeping all second coordination neighbors frozen, b) relaxation of the metal center keeping the first and second coordination neighbors frozen.

During our preliminary studies we relaxed a wide variety of clusters, requiring that the relaxed and non-relaxed geometries had as similar pair distribution profiles as possible. Indicatively relaxation of the first coordination shell inside an outer shell of frozen second closest neighbors invariably yielded spurious inter-atomic interactions. On the contrary, relaxation of just the metal centers in clusters frozen up to their second coordination shells provided good g(r) agreement with the un-relaxed geometries, both for charged and charge-neutral. Hence, all results shown henceforth are based on DFT relaxed centers within frozen nearest neighbors, inclusive of the second coordination shell.

**Fig. 4:**
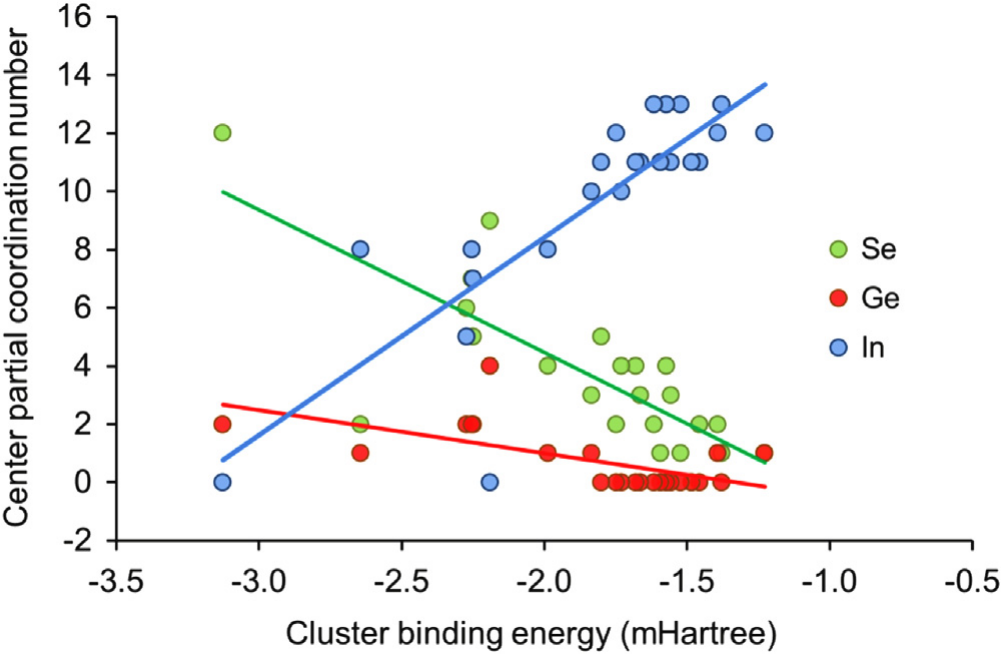
Cluster binding energy in respect to partial coordination of cluster centers, irrespective of central atomic species.

For all selected clusters it was then feasible to plot total coordination of the metal center in respect to DFT-calculated cluster binding energy, These results are depicted in Fig.4.

## 2 Conclusions

From the data, shown in Fig. 4 as the relationship between cluster binding energy and average coordination of cluster centers, a number of consistent conclusions were drawn

1. Indium content demoted Ge–Se bonding in favor of Se–In.
2. Cluster coordination by Se promoted stability while In coordination lowered cluster stability by interrupting the Ge–Se and Se–Se networks.
3. Ge–Se and Se–In bonding promoted overall cluster stability and the intervention of excess In caused breaking of these bonds contributed towards a lower binding energy.
4. On the whole, Ge and Se competed for connectivity with Se over the whole range of valence electron energies.

**Note on data files**

Data files underlying to this work are

1. RMC processed supercell files, GeSe4.wpd, GeSe4In5.wpd, GeSe4In10.wdp and GeSe4In15.wpd, Included in SupercellData.rar. These files contain both the cluster selection matrices in comma delimited format (see “Radial variation of coordination number, density and number density distributions” in each file, normalized as well as not normalized by the radial distance of the RMC simulation box) and the cluster coordinates (in xyz chemical format).
2. The individual (processed for pair correlation statistics) cluster files, included in ClusterData.rar.
3. The final results file, Results.xlsx, inclusive of Fig. 2 and the datasets that produced the latter.

## Figures and Tables

**Table 1:** The coordinates (coordination vs. distance from the supercell origin) of the peaks shown in Fig. 2 for the two case systems and the three supercell sizes for every system discussed. Each peak is characterized by a total coordination for Ge-, Se- and In-centered clusters and the distance between the center atom and the supercell origin. For example, in the case of the GeSe_4_In_10_ system, the coordination peak for Ge-centered clusters in the 3000 atom supercell (see Fig. 2a) involved coordination numbers of 15 and 16 and occurred for clusters 18 Å away from the supercell origin; the feature is denoted by ‘15,16/18’ in the Table. It is noted that all distances between the cluster centers and the supercell origins have been rounded up to the closest integer, hence, e.g., 18 Å include all distances between 17.50 and 18.49 Å.

GeSe_4_In_10_	3000	6000	18,000
Ge	15,16/18	17/18	18/18
Se	15,16,17/18	16/18	18/18
In	16/18	16/18	15,16/18
GeSe_4_In_15_	3000	6000	18,000
Ge	19,20/16	20/12	17/14
Se	16/18	19/18	16/18
In	16/18	16/18	16/18
